# A Nation-Wide multicenter 10-year (1999-2008) retrospective clinical epidemiological study of female breast cancer in china

**DOI:** 10.1186/1471-2407-11-364

**Published:** 2011-08-22

**Authors:** Jing Li, Bao-Ning Zhang, Jin-Hu Fan, Yi Pang, Pin Zhang, Shu-Lian Wang, Shan Zheng, Bin Zhang, Hong-Jian Yang, Xiao-Ming Xie, Zhong-Hua Tang, Hui Li, Jia-Yuan Li, Jian-Jun He, You-Lin Qiao

**Affiliations:** 1 Dept. of Cancer Epidemiology, Cancer Institute & Hospital, Chinese Academy of Medical Sciences & Peking Union Medical College, 17 South Panjiayuan Lane, Beijing 100021, China; 2 Center of Breast Disease, Cancer Institute & Hospital, Chinese Academy of Medical Sciences & Peking Union Medical College, 17 South Panjiayuan Lane, Beijing 100021, China; 3 Dept. of Medical Oncology, Cancer Institute & Hospital, Chinese Academy of Medical Sciences & Peking Union Medical College, 17 South Panjiayuan Lane, Beijing 100021, China; 4 Dept. of Radiation Oncology, Cancer Institute & Hospital, Chinese Academy of Medical Sciences & Peking Union Medical College, 17 South Panjiayuan Lane, Beijing 100021, China; 5 Dept. of Pathology, Cancer Institute & Hospital, Chinese Academy of Medical Sciences & Peking Union Medical College, 17 South Panjiayuan Lane, Beijing 100021, China; 6 Dept. of Breast Surgery, Liaoning Cancer Hospital, No. 44 Xiaoyanhe Road, Dadong District, Shenyang 110042, China; 7 Dept. of Breast Surgery, Zhejiang Cancer Hospital, No. 38 Banshanqiao Guanji Road, Hangzhou 310022, China; 8 Dept. of Breast Oncology, Sun Yat-Sen University Cancer Center, 651 Dongfeng East, Gungzhou 510060, China; 9 Dept. of Breast-thyroid Surgery, Xiangya Sencod Hospital, Central South University, No. 139 Renminzhonglu, Changsha 410011, China; 10 Dept. of Breast Surgery, the Second People's Hospital of Sichuan Province, Chengdu 610041, China; 11 Dept. of Epidemiology, West China School of Public Health, Sichuan University, Chengdu, Sichuan 610041, China; 12 Dept. of Oncosurgery, the First Affiliated Hospital of Medical College, Xi'an JiaoTong University, 277 Yanta West Road, Xi'an 710061, China

## Abstract

**Background:**

According to the very limited cancer registry, incidence and mortality rates for female breast cancer in China are regarded to be increasing especially in the metropolitan areas. Representative data on the breast cancer profile of Chinese women and its time trend over years are relatively rare. The aims of the current study are to illustrate the breast cancer profile of Chinese women in time span and to explore the current treatment approaches to female breast cancer.

**Methods:**

This was a hospital-based nation-wide and multi-center retrospective study of female primary breast cancer cases. China was divided into 7 regions according to the geographic distribution; from each region, one tertiary hospital was selected. With the exception of January and February, one month was randomly selected to represent each year from year 1999 to 2008 at every hospital. All inpatient cases within the selected month were reviewed and related information was collected based on the designed case report form (CRF). The Cancer Hospital/Institute, Chinese Academy of Medical Sciences (CICAMS) was the leading hospital in this study.

**Results:**

Four-thousand two-hundred and eleven cases were randomly selected from the total pool of 45,200 patients and were included in the analysis. The mean age at diagnosis was 48.7 years (s.d. = 10.5 yrs) and breast cancer peaked in age group 40-49 yrs (38.6%). The most common subtype was infiltrating ductal carcinoma (86.5%). Clinical stage I & II accounted for 60.6% of 4,211 patients. Three-thousand five-hundred and thirty-four cases had estrogen receptor (ER) and progestin receptor (PR) tests, among them, 47.9% were positive for both. Two-thousand eight-hundred and forty-nine cases had human epidermal growth factor receptor 2(HER-2) tests, 25.8% of them were HER-2 positive. Among all treatment options, surgery (96.9% (4,078/4,211)) was predominant, followed by chemotherapy (81.4% (3,428/4,211). Much less patients underwent radiotherapy (22.6% (952/4,211)) and endocrine therapy (38.0% (1,599/4,211)).

**Conclusions:**

The younger age of breast cancer onset among Chinese women and more advanced tumor stages pose a great challenge. Adjuvant therapy, especially radiotherapy and endocrine therapy are of great unmet needs.

## Background

Breast cancer is the second most common cancer worldwide today, and by far the most common cancer in women in many countries, with an estimated 1.4 million new cases and 458,000 deaths around 2008 annually [1]. Although breast cancer incidence and mortality rates in the western countries have been decreasing or stable during the past 2 to 3 decades, both rates have been increasing rapidly in many developing countries [2]. In China, according to the most updated but limited cancer registries, breast cancer became the most important incident female cancer and ranked the 6^th ^leading cause of death in Chinese women in year 2006 [3]. No nation-wide representative data of breast cancer in China was available.

The etiology of breast cancer remains unknown. Due to the lack of evidence, primary preventive strategies for breast cancer have commanded little attention. Rather, the focus has been on improving prognosis (outcome) for women developing breast cancer, through early detection and improved treatment (surgical, radiotherapy, hormonal and chemotherapeutic measures). Current therapeutic options for breast cancer in China are varied, with treatment allocation generally dependent on the accessible resources, the patient's economic status and the tumor burden. In the recent decades, when estrogen receptor (ER) status was approved to be an important treatment and prognostic factor, targeted therapy has become encouraging in breast cancer treatment [4]. But due to limited access of updated guidelines and resources, information regarding the application of breast cancer treatment with this new therapy within China is not clear.

The nation-wide multi-center 10-year (1999-2008) retrospective clinical epidemiological study of female breast Cancer in China was a retrospective study of patients with pathology confirmed primary breast cancer from 7 geographic regions across China (North, North-East, Central, South, East, North-West, and South-West). The aims of this study were to document (1) the sociodemographic characteristics and the distribution of some risk factors among Chinese female breast cancer cases; (2) the clinical characteristics of female breast cancer and (3) current treatment options for Chinese female breast cancer patients.

## Methods

### Study Design

This study was a hospital-based multi-center 10 year retrospective study of randomly selected pathology confirmed primary female breast cancer cases via medical chart review.

### Selection of Regions and Hospitals

As described previously, China was stratified into 7 geographic regions according to the traditional administrative district definition; these regions extend over the majority of the country and represent different levels of breast cancer burden. One tertiary hospital from each region was selected to provide the required study cases.

**Figure 1:**
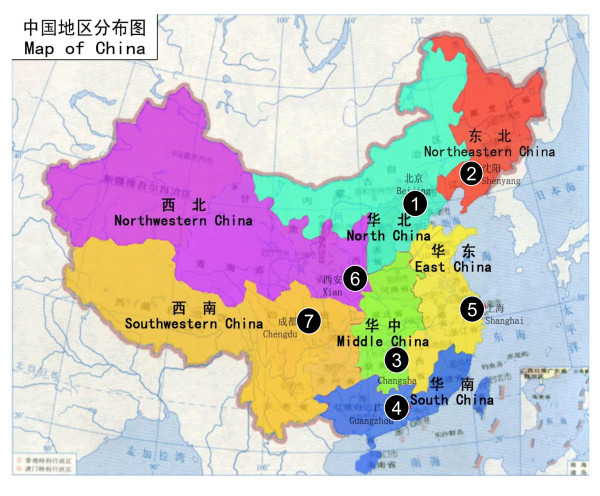
**Geographic distribution of sites included in the study**. 1: Cancer Institute/ Hospital, Chinese Academy of Medical Sciences 2: Liaoning Cancer Hospital 3: Second Xiangva Hospital, Central South University 4: Guangdong Sun Yat-Sen University Cancer Center 5: Zhejiang Cancer Hospital 6: First Affiliated Hospital of Xi'an Jiaotong University 7: Sichuan Cancer Hospital.

Convenient sampling was used to choose the participant hospitals with inclusion in the study on the basis that (1) they were one of the best leading hospitals at the tertiary level and had regional referral centers providing pathology diagnosis, surgery, radiotherapy, medical oncology, and routine follow-up care for patients with breast cancer; (2) visiting patients were from different parts of the region; and (3) the breast cancer screening practices, when used, should be in accordance with Chinese national standards. A total of 7 hospitals were involved in the study, with Cancer Hospital/Institute, Chinese Academy of Medical Sciences (CICAMS) as the lead center for the overall coordination of this research in China [Figure 1].

### Patients

This study included pathology confirmed female primary breast cancer inpatients in one randomly selected month each from year 1999 to 2008. January and February were excluded for randomization because Chinese traditional spring festival is always in these two months and there are much fewer inpatients during the time period. In order to avoid selection bias, an enrolment scheme was adopted using alternating prespecified month of inpatient admission from year to year. For example, in the first year (1999) of data collection, pathology confirmed primary breast cancer patients admitted to inpatient treatment in March would be enrolled into the study; in the second year (2000), inpatients admitted in April would be enrolled. All cases within the selected month were reviewed and patient's information was collected based on the designed case report form (CRF). In each selected month, if inpatients admissions were less than 50 in that year, more cases from the neighbouring months were reviewed until the total number in that year reaches 50. Whereas, if inpatients number in the selected month exceeded 50, all cases should be reviewed. To ensure that the national study was geographically representative, it was designed to include patients enrolled at sites from all 7 traditional regions across China [Figure 1].

All patients enrolled in this study must meet 3 key inclusion criteria: (1) pathology confirmed primary breast cancer (2) inpatient admission date was within the selected month in the study hospital and (3) received or receiving treatment (surgery, medical oncology, radiotherapy) for breast cancer.

This study was approved by the Cancer Foundation of China Institutional Review Board. Patient consent was not required for this study because there were no anticipated risks for the participants of the study. The data was stripped of any patient identifiers per the approved procedures. De-identified data were maintained on secure database. Only research team members have access to the data. All data will be reported in aggregate.

### Pathologic Diagnostic Criteria

Histological subtype was based on the 1981 and 2003 WHO histological classification criteria [5,6]. Staging of breast cancer was done according to the AJCC TNM staging system of year 1997 and after [7,8].

### Data Collection and Quality Control

The following data were systematically collected for all enrolled patients via medical chart review: (1) general information including date of diagnosis, visits to other health care professionals, inpatient admission date, diagnosis at admission, inpatient discharge date, and discharge outcome; (2) demographic characteristics at the time of diagnosis/admissions including age, occupation, height, weight, education and marital status; (3) breast cancer risk factors such as age at menarche, age at menopause, age at marriage, age at first delivery, number of live birth, breastfeeding history, family history of breast cancer, use of oral contraception, history of smoking and alcohol drinking; (4) results of the clinical breast examination (CBE); (5) results of the diagnostic imaging including mammography, ultrasound and magnetic resonance imaging (MRI); (6) the use of currently available surgery approaches; (7) the use of radiotherapy for breast cancer; (8) the use of chemotherapy for breast cancer, including adjuvant chemotherapy and neoadjuvant chemotherapy; (9) the use of molecular targeted therapy for breast cancer; (10) pathological characteristics including pre-surgery cytology and pathologic examinations, intraoperative pathologic evaluation, post-surgery pathology, estrogen and progesterone receptor expressions and human epidermal growth factor receptor 2(HER-2) expression etc. Also, the ranking and number of the 10 most dominant cancer patients visits were also collected.

All above information was extracted from medical charts to the designed CRF by local clerks after training. Then two data input clerks from each site were recruited to independently double-enter data from the paper to computer based database (FoxPro). All finished double-entry databases were sent to CICAMS for validation by running EpiData. Any inconsistency found by CICAMS between the two databases was reported to the local clerks for adjudication until the databases agreed. As a final inspection, one of databases was chosen to undergo a final consistency check. Logical errors (e.g. a woman had no surgery yet had intraoperative frozen section diagnosis) were again reported back to the local sites, and the local collaborators reviewed the original medical chart again. After checking with the original medical record, the local staff sent the revised database back to CICAMS for a final analysis. During the consistency check, 5% of the medical charts were randomly selected based on the study ID and sent to CICAMS for quality control review.

### Sample Size

Sample size requirements for the Chinese regional sites were a minimum of 50 patients per site per year between 1999 and 2008. To capture treatment and clinical outcomes in breast cancer over 10 years, it was mandatory to collect a total number of no less than 500 cases per hospital in time span. Pooling of data across all sites in China was employed to adequately describe disease and treatment characteristics across the country.

### Data Analysis

Frequencies were run on variables related to demographic, reproductive, clinical and pathologic characteristics to determine their distribution overall and among early stage (ES) (stage I & II) compared to late stage (LS) (stage III & IV). Quantitative variables were calculated by the mean or median depending on the data distribution. The differences in distribution of variables between ES and LS were examined using Mantel-Haenszel chi-square tests and Fisher's exact tests to obtain *p*-values for the test of no-association.

SPSS statistical software version 17.0 was used to analyze the data. Statistical significance was assessed by two-tailed tests with α level of 0.05.

## Results

A total number of 4,267 breast cancer inpatients across the various geographic regions in the study were collected. Of them, 48 cases were excluded for analysis because they were not from the selected months. Seven were deleted because the final pathological diagnoses belonged to benign tumors instead of malignancy. One case was disqualified because the 'unknown' items exceeded 50% of the CRF. A final total number of 4,211 cases from seven sites across China were included for final analysis. Table 1 illustrates the case distribution and proportion by region and year.

**Table 1:** Case distribution by region and year

Year	North		North-East		Central		South		East		North-West		South-West		Total	
								
	N*	n**(%)	N*	n**(%)	N*	n**(%)	N*	n**(%)	N*	n**(%)	N*	n**(%)	N*	n**(%)	N*	n**(%)
**2008**	1649	91(5.5)	1632	82(5.0)	647	73(11.3)	782	75(9.6)	1400	81(5.8)	894	45(5.0)	508	50(9.8)	7512	497(6.6)
**2007**	1390	110(7.9)	1486	134(9.3)	532	66(12.4)	835	74(8.9)	1262	83(6.6)	458	51(11.1)	404	50(12.4)	6367	568(8.9)
**2006**	1208	87(7.2)	1377	95(6.9)	368	60(16.3)	781	53(6.8)	1144	68(5.9)	215	49(22.8)	357	50(14.0)	5450	462(8.5)
**2005**	1108	60(5.4)	1195	75(6.3)	330	55(16.7)	734	59(8.0)	1008	58(5.8)	465	49(10.5)	356	50(14.0)	5196	406(7.8)
**2004**	902	50(5.5)	1152	96(8.3)	340	51(15.0)	620	53(8.6)	1065	64(6.1)	313	53(16.9)	322	50(15.5)	4714	417(8.9)
**2003**	715	50(7.0)	1097	78(7.1)	294	49(16.7)	527	59(11.2)	875	52(5.9)	179	53(29.6)	265	49(18.5)	3952	390(9.9)
**2002**	764	41(5.4)	1044	51(4.9)	227	46(20.3)	397	58(14.6)	593	51(8.6)	157	44(28.0)	207	50(24.2)	3389	341(10.1)
**2001**	681	52(7.6)	982	74(7.5)	144	46(31.9)	374	58(15.5)	545	50(9.2)	153	47(30.7)	380	50(13.2)	3259	377(11.6)
**2000**	587	50(8.5)	874	52(6.0)	106	48(45.3)	289	55(19.0)	461	50(10.9)	136	45(33.1)	318	50(15.7)	2771	350(12.6)
**1999**	532	50(9.4)	831	95(11.4)	139	52(37.4)	298	60(20.1)	392	49(12.5)	156	47(30.1)	242	50(20.7)	2590	403(15.6)
**Total**	9536	641(6.7)	11670	832(7.1)	3127	546(17.5)	5637	604(10.7)	8745	606(6.9)	3126	483(15.5)	3359	499(14.9)	45200	4211(9.3)

N* number of the actual breast cancer visits each year from year 1999-2008;

n** number of breast cancer cases collected per protocol.

### Patients Characteristics

Of the total 4,211 cases that were identified over a period of 10 years from 1999 to 2008, the mean age at diagnosis and age range for all breast cancer patients was 48.7 years (s.d. = 10.5 yrs) and 21-86 years, respectively. Among all cases, 2,554 (60.6%) were in ES with a mean age of 49.1 years (s.d. = 10.4 yrs) while 901 (21.4%) were in LS with a mean age of 48.8 years (s.d. = 10.3 yrs). The majority (67.6%) of breast cancer patients were of normal Body Mass Index (BMI). More breast cancer cases presented in LS (59.6%) were manual workers (*P *< 0.001) and the proportion of receiving higher education (university and above) was much less compared with the ES group (14.2% vs. 21.3%, *P *= 0.008). The percentage distribution of smoking, alcohol drinking and family history of breast cancer differed between women presented in ES and LS (*P *< 0.050) (Table 2). More breast cancer cases (7.6% vs. 6.3%. *P *= 0.030) presented in LS reported to have the first delivery at or older than 30 years of age and the percentage distribution of self reported oral contraceptive use differed between women presented in ES and LS (*P *< 0.001) (Table 3).

**Table 2:** General characteristics of breast cancer cases

Variables by level	Total distribution		Clinical Stage I&II		Clinical Stage III&IV		ES vs. LS *P*-value
	(N = 4211)		(N = 2554)		(N = 901)		
		
	n	%	n	%	n	%	
**Age at Diagnosis (Years)** **(4211, 2554, 901)**							
Mean ± SD	48.7 ± 10.5	--	49.1 ± 10.4	--	48.8 ± 10.3	--	0.345^a^
Range	21~86	--	21~86	--	21~85	--	
≤ 29	80	1.9	38	1.5	17	1.9	
30-39	710	16.9	404	15.8	144	16.0	
40-49	1624	38.6	987	38.7	341	37.9	0.088^b^
50-59	1147	27.2	693	27.1	276	30.6	
≥ 60	650	15.4	432	16.9	123	13.7	
**Body Mass Index** **(3281, 2107, 646 )**							
Mean ± SD	23.4 ± 3.3	--	23.3 ± 3.2	--	23.5 ± 3.4	--	0.173^a^
Range	12.8~39.4	--	14.7~36.1	--	12.8~39.4	--	
Underweight (≤ 18.49)	140	4.3	74	3.5	30	4.6	
Normal Weight (18.50~24.99)	2217	67.6	1461	69.3	424	65.6	0.196^b^
Overweight (25.00~29.99)	818	24.9	511	24.3	167	25.9	
Obesity (≥ 30.00)	106	3.2	61	2.9	25	3.9	
**Occupation (3623, 2192, 796)**							
Housewife	173	4.8	127	5.8	26	3.3	
Manual Worker	1893	52.3	1099	50.1	474	59.6	< 0.001^b^
Mental Worker	1028	28.4	659	30.1	189	23.7	
Others	529	14.6	307	14.0	107	13.4	
**Education (2091, 1315, 486)**							
None	186	8.9	112	8.5	55	11.3	
Primary School	462	22.1	273	20.8	115	23.7	
Middle School	606	29.0	370	28.1	141	29.0	0.008^b^
High School	441	21.1	280	21.3	106	21.8	
University and above	396	18.9	280	21.3	69	14.2	
**Marital Status (4193, 2548, 899)**							
Single	51	1.2	25	1.0	14	1.6	
Married	4090	97.5	2482	97.4	878	97.7	0.073^b^
Widowed/Divorced	52	1.2	41	1.6	7	0.8	
Region (4211, 2554, 901)*							
More Developed	1851	44.0	1127	44.1	389	43.2	0.620^b^
Less Developed	2360	56.0	1427	55.9	512	56.8	
**Smoking History (4211, 2554, 901)**							
Never	2468	58.6	1451	56.8	596	66.2	
Used	47	1.1	37	1.5	9	1.0	< 0.001^c^
current	12	0.3	5	0.2	3	0.3	
Unknown	1684	40.0	1061	41.5	293	32.5	
**Alcohol Drinking History** **(4211, 2554, 901)**							
Never	2427	57.6	1415	55.4	598	66.4	
Used	82	2.0	70	2.7	6	0.7	< 0.001^c^
Now	13	0.3	7	0.3	4	0.4	
Unknown	1689	40.1	1062	41.6	293	32.5	
**Breast Cancer Family History (4128, 2517, 882)**							
Yes	144	3.5	96	3.8	21	2.4	0.045^b^
No	3984	96.5	2421	96.2	861	97.6	

^a ^Student's t-test

^b ^Chi-square test

^c ^Fisher's exact test

* More Developed Regions: North, South, and East

Less Developed Regions: North-East, Central, North-West, South-West.

**Table 3:** Reproductive characteristics of breast cancer cases

Variables by level	Total distribution (N = 4211)		Clinical Stage I&II (N = 2554)		Clinical Stage III&IV (N = 901)		ES vs. LS *P*-value
	n	%	n	%	n	%	
		
							
**Menopausal Status** **(4211, 2554, 901)**							
Pre-menopausal	2649	62.9	1585	62.1	541	60.0	0.285^b^
Post-menopausal	1562	37.1	969	37.9	360	40.0	
**Age at Menopause (Years)** (1562, 969, 360) **							
≤ 50	1017	65.1	631	65.1	239	66.4	0.665^b^
> 50	545	34.9	338	34.9	121	33.6	
**Age at Menarche (Years)** **(150, 80, 50)**							
7~11	3	2.0	0	0.0	2	4.0	
12~13	37	24.7	22	27.5	11	22.0	0.200^c^
≥ 14	110	73.3	58	72.5	37	74.0	
**Age at First Delivery (Years)** **(2021, 1372, 357 )**							
< 20	33	1.6	15	1.1	11	3.1	
20~24	917	45.4	638	46.5	153	42.9	0.030^b^
25~29	940	46.5	632	46.1	166	46.5	
≥ 30	131	6.5	87	6.3	27	7.6	
**Number of Live Births** **(3947, 2428, 807)**							
0	99	2.5	44	1.8	17	2.1	
1	1889	47.9	1177	48.5	348	43.1	
2	1228	31.1	767	31.6	269	33.3	0.082^b^
3	452	11.5	271	11.2	110	13.6	
≥ 4	279	7.1	169	7.0	63	7.8	
**Breast Feeding History** **(2671, 1754, 492)**							
Yes	2414	90.4	1591	90.7	445	90.5	0.861^b^
No	257	9.6	163	9.3	47	9.5	
**O.C Consumption History** **(4211, 2554, 901)**							
Never	543	12.9	376	14.7	98	10.9	
Used	325	7.7	278	10.9	36	4.0	< 0.001^c^
Now	11	0.3	10	0.4	1	0.1	
Unknown	3332	79.1	1890	74.0	766	85.0	

^a ^Student's t-test

^b ^Chi-square test

^c ^Fisher's exact test

** Median age of menopause has been used to create the two age-categories.

Table 4 illustrates the clinical and pathologic characteristics of patients. The average mass size by CBE was 31.3 mm (s.d. = 17.8 mm) and patients in ES were more likely to be examined of having masses less than 50 mm (*P *< 0.001) and had a much lower chance of having local invasion (*P *< 0.001). Patients in LS were more likely to have positive mammography results (*P *= 0.001) and positive ultrasound diagnosis (*P *= 0.015). Over the 10 years, invasive ductal carcinoma remained the dominant pathologic subtype (86.5%). Among the 3,534 patients that had estrogen receptor (ER) and progesterone receptor (PR) information, less than 10% (9.5%) were only ER positive and PR negative; 10.4% were positive with PR but negative with ER; about half (47.9%) were both ER and PR positive and 32.2% were negative with both. HER-2 information was available for 2,849 patients and the majority (74.2%) of them were HER-2 negative (Table 4). Among the 4,211 cases, the majority (60.6% (2,554/4,211)) had ES breast cancer, and less than a quarter (21.4% (901/4,211)) had LS disease. About 18% (756/4,211) of cases did not have staging information. The stage distribution differed between the less developed and more developed regions of China (*P *< 0.001). More stage I patients (19.9%) were presented in more developed regions than that (12.7%) in less developed regions and the reverse was seen for stage IV patients (Table 5).

**Table 4:** Clinical and pathologic characteristics of breast cancer cases

Variables by level	Total distribution		Clinical Stage I&II		Clinical Stage III&IV		ES vs.LS *P*-value
	(N = 4211)		(N = 2554)		(N = 901)		
		
	n	%	n	%	n	%	
**Palpable Tumor Location by CBE(4211, 2554, 901)**							
Left	2189	52.0	1327	52.0	477	52.9	
Right	1995	47.4	1213	47.5	419	46.5	0.868^c^
Non-palpable	27	0.6	14	0.5	5	0.6	
**Mass Size by CBE** **(3555, 2434, 825)**							
Mean ± SD	31.3 ± 17.8	--	27.7 ± 12.8	--	40.7 ± 23.4	--	< 0.001^a^
Range	0~250	--	0~150	--	0~250	--	
< 50 mm	3036	85.4	2247	92.3	559	67.8	
≥ 50 mm	519	14.6	187*	7.7	266	32.2	< 0.001^b^
**Local Invasion** **(3663, 2428, 826)**							
Yes	183	5.0	45	1.9	106	12.8	< 0.001^b^
No	3480	95.0	2383	98.1	720	87.2	
Mammography Diagnosis (1275, 877, 286)**							
Positive	1094	85.8	750	85.5	266	93.0	0.001^b^
Negative	181	14.2	127	14.5	20	7.0	
Ultrasound Diagnosis (2253, 1629, 409 )**							
Positive	2006	89.0	1461	89.7	383	93.6	0.015^b^
Negative	247	11.0	168	10.3	26	6.4	
**Postoperative Pathological Diagnosis (4014, 2540, 877)**							
Carcinoma in Situ(CIS)	143	3.6	77	3.0	5	0.6	
Invasive Ductal Carcinoma	3471	86.5	2203	86.7	801	91.3	< 0.001^c^
Other Type Invasive Carcinoma	385	9.6	252	9.9	69	7.9	
Others	15	0.3	8	0.3	2	0.2	
**ER/PR Status** **(3534, 2316, 758)**							
ER+&PR+	1691	47.9	1148	49.6	340	44.9	
ER+&PR-	337	9.5	204	8.8	84	11.1	0.070^b^
ER-&PR+	367	10.4	230	9.9	86	11.4	
ER-&PR-	1139	32.2	734	31.7	248	32.7	
**HER-2 Status** **(2849, 1935, 582)**							
HER-2 +	736	25.8	504	26.0	154	26.5	0.842^b^
HER-2 -	2113	74.2	1431	74.0	428	73.5	

^a ^Student's t-test

^b ^Chi-square test

^c ^Fisher's exact test

* Among the 187 women with palpable lumps ≥ 50 mm by CBE, 101 were found to have smaller lumps based on the postoperative evaluation and were categorized as stage I or stage IIA (16 as stage I; 85 as stage IIA). The rest 86 were categorized as stage IIB.

** Negative: negative, benign, and possibly benign finding; Positive: malignant suspicious, malignant.

**Table 5:** Stage distribution of breast cancer cases by region

Clinical Stage	Total		More Developed Regions**								Less Developed Regions***										P*
					
			North		South		East		Sub-total		Northeast		Central		North-West		South-West		Sub-total		
	N	%	N	%	N	%	N	%	N	%	N	%	N	%	N	%	N	%	N	%	
Stage I	663	15.7	172	26.8	98	16.2	95	15.7	365	19.9	131	15.8	73	13.4	77	15.9	17	3.4	298	12.7	
Stage II	1891	44.9	280	43.7	243	40.2	239	39.4	762	41.6	323	38.8	400	73.3	170	35.2	236	47.3	1129	48.3	
Stage III	788	18.7	93	14.5	117	19.4	152	25.1	362	19.8	119	14.3	53	9.7	127	26.3	127	25.5	426	18.2	< 0.001
Stage IV	113	2.7	2	0.3	20	3.3	5	0.8	27	1.5	4	0.5	3	0.6	56	11.6	23	4.6	86	3.7	
Unknown	756	18.0	94	14.7	126	20.9	115	19.0	335	18.3	255	30.7	17	3.1	53	11.0	96	19.2	421	18.0	

* Stage distribution in more developed regions vs. less developed regions

** More Developed Regions: North, South, and East

***Less Developed Regions: Northeast, Central, North-West, South-West.

### Treatment Patterns

Among all breast cancer cases, the majority (96.9% (4,078/4,211)) had undergone surgery procedures and radical mastectomy was the predominant option (88.8% (3,740/4,211). A minority of women (5.5% (231/4,211)) received breast conservative surgery. Chemotherapy was the second most important treatment option (81.4% (3,428/4,211) for breast cancer patients in China. By comparison, radiotherapy (22.6% (952/4,211)) and endocrine therapy (38.0% (1,599/4,211)) were not as popular as surgery and chemotherapy (Table 6).

**Table 6:** Treatment patterns of breast cancer cases

Therapy	Total	
	(N = 4211)	
	n	%
**Surgery**		
No	107	2.5
Radical Mastectomy	3740	88.8
Breast Conservative Surgery	231	5.5
Simple Mastectomy	46	1.1
Others	61	1.5
Unknown	26	0.6
**Radiotherapy**		
No	2723	64.7
Yes	952	22.6
Unknown	536	12.7
**Chemotherapy**		
No	626	14.9
Yes	3428	81.4
Unknown	157	3.7
**Endocrine Therapy**		
No	2092	49.7
Yes	1599	38.0
Unknown	520	12.4

## Discussion

This was the first geographically representative epidemiologic study of breast cancer in China and included more than 4,000 patients over its course. This study, via its inclusion of a large number of sites across all seven traditional regions of China, facilitated a thorough assessment of breast cancer patient characteristics, treatment allocation, and allowing a unique analysis of possible regional variations in these aspects of breast epidemiology and management across the entire country. It also helps to determine levels of unmet medical need and identify regions of high risk for breast cancer within China.

All patients included in our study were ethnically Chinese. Their clinical characteristics were significantly different from those of the women in western countries. The mean age at diagnosis was 48.7 years, and this was similar to the findings from other regional studies within China [9-14]. It was also in agreement with reports from other Asian countries such as Singapore, India and also similar to Saudi Arabia, all of which were around the mid-40s. This was about a decade earlier than what is reported for Western Caucasian women [15-19]. The reasons for the distinctions remain obscure, but four hypotheses may explain. First, older Asians including Chinese women had been less exposed to estrogen related risk factors thus have been less susceptible to breast cancer than their younger counterparts. Second, younger women were more genetically predisposed to breast cancer. Third, younger women were more aware of breast cancer. They have broader access to medical care as there is no nationwide organized screening program in China as well as in the majority of the modernizing Asian countries. Fourth, mammography has been used among older women in the population based mammography screening in Western countries. This may partly account for why breast cancer clusters peak around 60-69 years in Western countries.

In this study, 60.6% of the patients had early stage breast cancer and 21.4%% had late stage disease. The incidence of early stage disease on presentation was lower than the data from China Tianjin (72.3%) [14], Taiwan (78.3%) [13] and Singapore (79%) [20], and much lower than the western countries such as the United States (85%) [21]. A study from Hong Kong which focused on a selected group of affluent Chinese patients reported an 88.9% of early stage cases [19]. While in the African countries, large proportions of patients presenting at late stage were reported, early stage cases accounted for only 9.27% to 42.7% [22-25]. The vast difference between regions and countries may due to the absence of a nationwide breast cancer screening program in developing countries including China, whereas such programs are fully or partly implemented in the majority of developed countries [26]. The findings of our study also suggest that women who were mental workers and had at least a university education were more likely to present breast cancer at early stage. This is in consistent with reports from a review article that socioeconomic disparities including low family income, poor educational attainment and impaired access to healthcare etc were related to the more advanced disease at diagnosis and poorer prognosis [27].

ER positive breast cancers are acknowledged to be related to a better prognosis than those that are ER negative [28] as they respond better to hormone therapy [29]. HER-2 positive breast cancers are more aggressive and require more expensive therapy [30]. In our study, 57.4% (2,028/3,534) were ER positive and 25.8% (736/2,849) were HER-2 positive. The ER status of Chinese breast cancer was documented previously and the positivity varied from 45.3% to 67% [31-35]. When compared with data from developed countries, the positivity from our study is significantly lower [36,37]. The prevalence of HER-2 has been documented to be 27.9% in a Chinese study [35] and 15% in one study from the United States [38]. It suggests that breast cancer in Chinese women may be more aggressive than those in the developed countries, but those differences may also be explained by the un-uniformed tests used, different cut-off value referred, and bias from the age distribution in various studies. Although our study sample is representative, the tests were done retrospectively and different methods and protocols were conducted. Further study using a representative sample and standard protocol to understand the status of ER/PR/HER-2 status in Chinese breast cancer is necessary and would make it more comparable.

Surgery was the most common treatment in Chinese female breast cancer patients followed by chemotherapy. Among all surgery procedures, radical mastectomy was widely perceived as the only curative treatment, which is consistent with a study from Hong Kong [39]. Options for radiotherapy and endocrine therapy were much less, which indicates that adjuvant therapy, especially radiotherapy and endocrine therapy are of great unmet needs. Further analysis and studies were necessary to understand the patterns of treatment based on detailed information of treatment indications such as tumor size, lymph node involvement, final margins, and ER status.

These findings will need to be considered in light of the study's strengths and weaknesses. The primary strengths of this study are (1) the large number of patients included and (2) the geographic representativeness of the included sites. The main potential study limitations are (1) selection bias may exist in the catchment of breast cancer patients in the selected hospitals as no less elite hospitals as comparison were selected from the same regions. (2) There is no comparison group to compare the risk factors of developing breast cancer and (3) data quality is dependent on the thoroughness of the clinician's documentation of medical history, treatment, and outcomes.

## Conclusion

The Chinese breast cancer multi-center clinical epidemiologic study represents the first geographically representative study of breast cancer in China to understand patterns of breast cancer characteristics, therapy use and knowledge of continuing unmet needs for breast cancer by retrospectively reviewing the existed clinical data.

The younger age of breast cancer onset among Chinese women and more advanced tumor stages pose a great challenge for Chinese government on breast cancer control. The higher proportion of ER+/PR+ breast cancer patients is a great challenge for breast cancer management in China. The Government oriented campaign to raise awareness will need to be expanded and continued. The most updated clinical guideline will also need to be disseminated to doctors at all levels to benefit the patients, ultimately improving the prognosis in Chinese breast cancer patients.

## List of abbreviations

CICAMS: Chinese Academy of Medical Sciences; CRF: Case Report Form; CBE: Clinical Breast Examination; MRI: Magnetic Resonance Imaging; ES: Early Stage; LS: Late Stage; ER: Estrogen Receptor; PR: Progestin Receptor; HER-2: Human Epidermal growth factor Receptor 2; BMI: Body Mass Index; O.C: Oral Contraceptives.

## Competing interests

The authors declare that they have no competing interests.

## Authors' contributions

JL helped to design, analyze, and interpret the data. She also drafted the initial manuscript.

BNZ helped to design the study and is the clinical PI of the study. JHF and YP helped with the data management and analysis. PZ, SLW and SZ helped design the CRF to collect the data. BZ, HJY, XMX, ZHT, HL, JYL, and JJH were the local PIs that helped with data collection. YLQ was the PI of this study and assisted in designing the study and performed critical revisions of the manuscript. All authors read and approved the final manuscript.

## Pre-publication history

The pre-publication history for this paper can be accessed here:

http://www.biomedcentral.com/1471-2407/11/364/prepub
